# Validating a Child Youth Resilience Measurement (CYRM-28) for Adolescents Living With HIV (ALHIV) in Urban Malawi

**DOI:** 10.3389/fpsyg.2020.01896

**Published:** 2020-08-31

**Authors:** Blessings N. Kaunda-Khangamwa, Innocent Maposa, Rosalia Dambe, Kennedy Malisita, Emmanuel Mtagalume, Lalio Chigaru, Alister Munthali, Effie Chipeta, Sam Phiri, Lenore Manderson

**Affiliations:** ^1^School of Public Health, University of the Witwatersrand, Johannesburg, Johannesburg, South Africa; ^2^School of Public Health and Family Medicine, College of Medicine, University of Malawi, Blantyre, Malawi; ^3^Malaria Alert Centre, College of Medicine, University of Malawi, Blantyre, Malawi; ^4^Umodzi Family Centre, Blantyre, Malawi; ^5^Centre for Social Research, Chancellor College, University of Malawi, Zomba, Malawi; ^6^Centre for Reproductive Health, College of Medicine, University of Malawi, Blantyre, Malawi; ^7^Lighthouse Trust, Lilongwe, Malawi; ^8^School of Social Sciences, Monash University, Clayton, VIC, Australia; ^9^Institute at Brown for Environment and Society, Brown University, Providence, RI, United States

**Keywords:** Malawi, adolescents living with HIV, mixed methods, psychometric properties, resilience measures, teen-club clinic, validation

## Abstract

Resilience as a strength-based notion, measured across cultures, age groups, and sub-populations, contributes to understanding health and well-being. Yet, there is limited evidence of how the construct performs in resource-limited countries. We explored the psychometric properties of the CYRM-28 and validated the scale with adolescents living with HIV (ALHIV), a key sub-population. The participants included members of an advisory panel and 406 ALHIV, aged 15–19 years, attending an antiretroviral therapy and teen-club clinic in Blantyre, Malawi. This study employed a mixed-method study using an exploratory sequential design. The advisory panel discussed the CYRM-28, and select ALHIV then translated it into Chichewa, pilot-tested it using cognitive interviews, and back-translated it for clarity and appropriateness. The resultant CYRM-28 was tested using a survey with purposefully selected ALHIV. The overall median score was 123, with an interquartile range of 110–130. Minimum and maximum scores were 53 and 140. Cronbach’s alpha of 0.863 and Kaiser–Meyer–Olkin measure (0.866) confirmed internal consistency and the sample size adequacy, respectively. Bartlett’s tests of sphericity (*p* < 0.001) informed factor analysis. Exploratory factor analysis determined possible dimensions of resilience and the sub-scales. The confirmatory factor analysis (CFA) confirmed the construct validity and supported a three-factor model consistent with the conceptualization of resilience as a multi-dimensional construct. Structural equation modeling was applied to perform CFA to measure model of resilience. Multiple fit indices showed a good fit for the model. The CYRM-28 has good internal consistency, test and re-test reliability, and moderate convergent validity which render it useful as a self-report resilience measure to inform and evaluate interventions for the health and well-being of adolescents in Malawi.

## Introduction

Resilience describes doing well despite adversities. Resilience can be understood along a continuum where risks and protective factors interact, with individuals trying to reduce the effects of challenges or threats. Ungar defines resilience as “the capacity of individuals to navigate their way to the psychological, social, cultural, and physical resources that sustain their well-being, and their capacity individually and collectively to negotiate for these resources to be provided in culturally meaningful ways” (2008, 225). This definition encompasses multiple socio-ecological factors that influence positive outcomes over time. These are individual (cognitive skills, self-management), relational (peer, family, community support), and contextual (education, health, social services) (Obrist et al., 2010). The inclusion of socio-ecological factors in this study is critical for a nuanced understanding of resilience.

Globally, resilience researchers have used a variety of instruments to capture self-reported accounts of social well-being and health. Windle et al. (2011) and Henley (2010) identify that the most frequently used resilience tests and scales are COPE Scale, Rosenberg Self Esteem Scale, The Connor–Davidson Resilience Scale (Li et al., 2015), Child Youth Resilience Measure (CYRM) Resilience Scale (RS) (Sagone and De Caroli, 2014), and Antonovsky’s Sense of Coherence Scale (SOC) (Windle et al., 2011). No scale or measurement is considered as superior, and there is a continuous debate on their feasibility, reliability, and validity in different settings (DeMichelis and Ferrari, 2016). However, the scales are biased toward personal constructs for adults and children in high-income countries, and there has been little work conducted in low- or middle-income settings (van Rensburg et al., 2017).

Studies on resilience with adolescents have predominantly focused on mental health, often with a single focus such as the role of peers, family, school, or community-based resources to improve both social and health outcomes (Moore et al., 2012). Ungar and Liebenberg (2011), however, discuss the significance of multiple formal and mandated interventions (mental health, education, child welfare, and the justice system) to foster resilience. Similarly, Betancourt et al. (2013) and van Breda and Theron, both working in sub-Sahara Africa, discuss the interplay between individuals and contexts, including access to resources, in understanding resilience (2013; 2018). The Resilience Research Centre^1^ recommends the use of CYRM-28 to capture contextual and cultural elements considered relevant to develop a valid measure of resilience (Ungar, 2016).

There have been several attempts globally to validate the CYRM-28, including in Canada (Liebenberg et al., 2012), New Zealand (Sanders et al., 2017), Australia (Langham et al., 2018), and South Africa (van Rensburg et al., 2019). In the global north, there is variability in the factor structure correlations and relationships that measure resilience among youths (Pritzker and Minter, 2014). In South Africa, studies have been conducted on the analysis of resilience as a culturally and contextually entrenched construct among Sesotho-speaking youths (Van Breda and Theron, 2018), street youths (Malindi, 2014), and adolescents living with HIV (ALHIV) in KwaZulu Natal, using a factor structure analysis on youth experiencing sexual risks (Govender et al., 2017; van Rensburg et al., 2019). The aforementioned studies offer a rigorous cross-cultural adaptation process and psychometric analysis to reduce bias and allow for comparability of scores across cultures or populations (Jia and van de Vijver, 2015). However, methodological limitations within the CYRM include failure to load on one factor or sub-samples, and over-reliance on exploratory factor analysis (EFA) rather than confirmatory factor analysis (CFA) for the purposes of validation (Ungar and Liebenberg, 2011; Liebenberg et al., 2012). In addition to methodological limitations, findings on unique factors such as *ubuntu* and *batho* (community interdependence) as a source of resilience warrant further inquiry to understand concepts underlying resilience measurements among adolescents (Van Breda and Theron, 2018).

Ensuring that CYRM-28 measures are appropriate for ALHIV in Malawi is critical to identify factors and processes that characterize resilience in key sub-populations. Literature on resilience and HIV has particularly been informed by USAID programs under PEPFAR (United States President’s Emergency Plan for AIDS Relief) (Fleischman and Peck, 2017). This includes the DREAMS (Determined, Resilient, Empowered, AIDS-free, Mentored, and Safe) initiative, which focused on reducing the incidence of HIV among adolescent girls and young women aged 15–24 (Brown et al., 2018; Saul et al., 2018). The DREAMS initiative centers on interventions that reduce the impact of structural drivers, such as HIV risk, poverty, gender inequality, sexual violence, and lack of education, to promote health and social outcomes among young women and male partners (Saul et al., 2018). Other studies include resourcing resilience through targeted social cash transfers to 13–22-year-olds, in Mchinji to improve school retention (Toska et al., 2016) and in Zomba district to reduce the risk of sexually transmitted infections, including HIV (Baird et al., 2012). These studies did not measure resilience *per se*, and so far there has been no assessment of the CYRM-28 factor structure among adolescents living with HIV in the country. An understanding of the resilience construct among this subpopulation will enhance the monitoring and development of relevant interventions to improve well-being and livelihoods of ALHIV.

### The CYRM-28 Factor Structure

Despite the value of using CYRM-28, there is increasing evidence on the conflicting results from factor analysis, showing variability on the structure and scores, including the underlying construct (Liebenberg et al., 2012, 2017; van Rensburg et al., 2015). In their work with youth in Canada, Rensburg and colleagues used a 5-point Likert-type scale with three sub-scales (individual, relational, and context), and a 28-item list with response options ranging from “not at all” to “a lot” (Liebenberg et al., 2012). On the other hand, a study with youth in New Zealand used four sub-scales and a 28-item list, with four measuring spirituality, seven items on individual resources, seven items on family resources, and ten items on social and cultural resources (Sanders et al., 2015). The South African structure of CYRM-28 used with Sesotho-speaking youths combined social/cultural and communal/spiritual as one sub-scale, with less emphasis on individual sub-scales. Hence, resilience measures vary across context, sub-populations, and socio-cultural groups, warranting further investigation on validation processes, results, and interpretation (Windle et al., 2011; Jia and van de Vijver, 2015).

## Materials and Methods

### Study Design

Data reported in this article derive from a non-interventional mixed-method study. The study employed an exploratory sequential design, with a two-phase method to inform and modify research tools. The study conformed to recommendations for using CYRM-28, which included employing a six-member advisory panel to inform research study processes, site-specific questions, and the adaptation of the scale for ALHIV in Malawi (Ungar, 2016). The original version of the CYRM was translated into Chichewa, the dominant language in the study setting, and this version was validated for survey use among ALHIV. Routine programmatic data from registers (attendance, gender, ages, in and out of care) from 2010 to 2019 informed the characteristics of the study participants and settings, and the discussion section of this article.

### Study Setting

The study was conducted among ALHIV attending an Adolescent Teen Club at the Umodzi Family Centre (UFC) within Queen Elizabeth Central Hospital (QECH) in Blantyre, the major commercial city of Malawi. The QECH serves as a referral hospital for the south-west zone of the country. Adolescents attending the teen club, described in the next section, come from urban, peri-urban, and rural areas surrounding Blantyre. All adolescents testing HIV positive from these areas are counseled and offered enrollment in the UFC and other health centers and facilities surrounding QECH. Adolescents who need hospitalization are admitted to in-patient wards and are referred to UFC for HIV testing, confirmatory tests, and support. Before an adolescent is discharged, he or she receives another counseling session and is invited to join UFC or to attend an antiretroviral therapy (ART) clinic close to their home. As of June 30, 2019, the UFC ART clinic registry had a total of 14,160 people, of whom 3500 were adolescents aged 10–24.

### The Teen Club Program

The UFC teen club program is a Lighthouse Trust, Youth Friendly Health Service initiative, managed by UFC and supported by bilateral partners. The club model was adapted from the United States Baylor College of Medicine International Pediatric AIDS Initiative (BIPAI) in partnership with the Malawi Ministry of Health (Baylor College of Medicine International Pediatric AIDS Initiative (BIPAI) 2012; MacKenzie et al., 2017). Adolescents aged 10–19 are eligible to participate in the club programs after disclosure which entails their acceptance of their HIV diagnosis, its implications, and their disclosure of this to significant others. Adolescents are prepared to transition out of the teen club program from around 20 years of age. Currently, teen club services adhere to a “test and treat” policy and provide differentiated service delivery in terms of service intensity, frequency, location, and health worker support. The emphasis is on a patient-centered care approach to HIV care (Prust et al., 2017; Chihana et al., 2018).

To strengthen the clinical, psychosocial, and resource supports, various activities are scheduled throughout the year. Lighthouse Trust manages these activities with support from the Ministry of Health, local non-governmental organizations, and PEPFAR under the Centers for Disease Control and Prevention (Phiri et al., 2017; Prust et al., 2017; Moody et al., 2018). The teen club is held on Saturdays and provides comprehensive programming for those aged 10–19 years under three pillars: (1) Clinical Care, including screening and management of opportunistic infections, ART monitoring including side effects and viral load, nutrition screening and treatment, TB screening and treatment, and referrals for specialized care; (2) Psychosocial Support: adherence counseling, peer support, mental health screening, coping with stigma and discrimination, livelihoods, and future planning; and (3) Sexual and Reproductive Health services: education and contraception administration (Kim et al., 2014; MacKenzie et al., 2017).

### Study Participants

The study involved ALHIV, attending the UFC ART and teen-club clinic during the study period, with signed informed consent from parents/caregivers and assent from those aged 15–17 years, and informed consent from those aged 18–19 years. The study pool purposefully selected young people aged 15–19 because of the continued disproportionate effects on this group from HIV and the only age group to have experienced a rise in deaths caused by AIDS-related illness (UNAIDS, 2019). Participants included those attending (or not) the ART clinic and UFC teen club, in and out of school, pregnant or not, and included both those born with HIV and those horizontally infected. People excluded from the study included those who were critically ill, and those aged below 15 or above 19 years.

### Sampling

**FIGURE 1:**
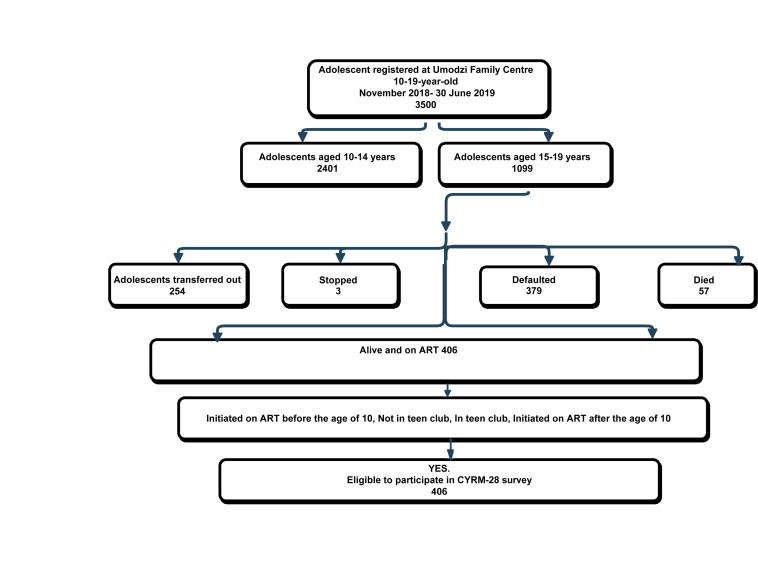
Summary of study sample.

Sampling was based on similar studies conducted with adolescents attending Saturday teen-club clinics in Zomba and Lilongwe, in Malawi by Kim et al. (2015, 2017) and MacKenzie et al. (2017). Through the electronic medical records, we purposefully selected adolescents aged 15–19 between January 2018 and June 2019 attending the UFC ART clinic. Through the UFC database, a total of 1099 ALHIV were identified and invited to participate in the study. Follow-up revealed that 254 had transferred out, 3 had stopped attending the clinic, 379 had defaulted, and 57 were reported dead by October 2018. A total of 406 ALHIV who had been on ART for the past 90 days were eligible to participate (Figure 1).

### Recruitment Processes

All adolescents attending the UFC ART and teen-club clinics were invited to take part in the study when they presented at the clinic reception. The support staff working and adolescents’ mentors at the ART clinic gave out letters of invitation written in vernacular, Chichewa. They explained briefly the study objectives to all adolescents aged 15–19, over an 8-month period (November 2018–June 2019). Adolescents were given a chance to be recruited as they would normally visit the clinic for routine follow-up and attend the teen club every month or two. There were two different letters of invitations for adolescents aged 15–17 to attend, with their parents signing the consent form while they signed an assent form; those aged 18 and 19 provided a written consent form.

### Instruments, Measures, and Procedures

#### Site-Specific Questions

Consistent with the CYRM manual regarding site-specific questions to capture context, relevant questions were conceptualized during the pilot survey and shared with the panel of six advisors as part of triangulation (Ungar, 2016). There were 11 questions, including statements on life experiences and adolescents’ knowledge of service use, for example: “Being an adolescent is challenging,” “I can handle difficulties in my life,” “I have knowledge of sexual and reproductive health,” and “the teen-club clinic provides support.” The 11 site-specific items were measured on a 5-point Likert scale, ranging from 1 (not at all) to 5 (a lot) (Appendix 1).

#### CYRM-28

The adapted version of the CYRM-28 (Liebenberg et al., 2012) was administered to measure ALHIV resilience. Resilience is a latent variable that comprises 28 items with eight components (individual: personal and social skills, peer support; caregiver physical and psychological and context: spiritual, education, and cultural), which provide insights to three subscales (Ungar, 2016). The individual sub-scale has 11 items that are divided under (1) personal skill with (5 items), peer support (2 items), and social skill (4 items); (2) the relational sub-scale considers physical and psychological caregiving with 2 and 5 items, respectively; and (3) the context sub-scale focuses on spirituality (3 items), education (2 items), and culture (5 items) (van Rensburg et al., 2017). The following are some examples of statements under individual personal skills – “I am able to solve problems without hurting myself or others”; caregiving – “My caregiver stands by me during difficult times”; context – “I enjoy my community’s traditions” (Ungar, 2016).

In this article, we concentrate on adaptation processes and changes to the wording of the CYRM-28 items. These modifications are consistent with the manual to ensure context relevance and familiarity with competing measures (van Rensburg et al., 2015, 2017). The items are measures on a 5-point Likert scale, ranging from 1 (not at all) to 5 (a lot) with higher scores (≤140) representing increased resilience (Ungar and Liebenberg, 2011). A summary of these 28 items within their sub-scales is set out in Appendix 2.

### Data Collection

Data collection occurred in two phases. The first phase was a pilot and pre-test study, using cognitive interviews with 42 participants to determine if ALHIV understood the questions and underlying concepts in the CYRM-28. The interviews were held in Zomba (70 km from Blantyre) at Tisungane teen club, Zomba Central Hospital, as a rapport-building activity; we targeted cognitive processes to ensure that the questions worked as intended (Zukerberg and Hess, 1996; Ungar, 2008).

Previous studies on cognitive interviewing have used “think aloud” and verbal probing techniques with a group of 5–10 subjects to discuss concepts and question-wording formats (Blair and Brick, 2010). In this study, we held the first three rounds of cognitive interviews among young people aged 15–16, 17–18, and 19 years. We employed verbal probing techniques with six adolescents in each groups. The remaining four rounds were spread over 3 weeks to follow up questions, probe on meanings of resilience, explore the reasons for varied responses, and gather insight on clarity of the questions to improve the English and Chichewa interview guides (Heather et al., 2012).

To minimize bias in both the pilot and post-pilot studies, the first author trained the same four research assistants on the purpose of the study, objectives, and processes to administer the survey. The research assistants were part of the mentorship program under UFC team and underwent three rounds of completing the CYRM form with feedback sessions to ensure standardized administration of the questionnaire (Jia and van de Vijver, 2015). In addition, weekly and monthly meetings were held with the data collection team to ensure consistency in the language used and instructions to complete the survey.

The pilot established a draft of context-specific questions, which incorporated the CYRM-28. This was presented and discussed with the advisory panel to ensure contextual relevance. Slight changes were made to the wording as all questions were translated into the vernacular to ensure that adolescents were able to understand the questions. A single survey questionnaire with the modified CYRM-28 was then drafted (Appendix 2).

The second phase of data collection took place with individuals and in groups in Blantyre from November 2018 to June 2019. The final questionnaire was administered to 406 adolescents from UFC ART and teen-club clinics in Blantyre. The questionnaire as described previously was structured and included both open- and close-ended and Likert-scale questions (Windle et al., 2011; van Rensburg et al., 2015).

The adolescents completed the paper-based questionnaire in a classroom set-up in the lecture hall at the College of Medicine through 20 group sittings (9–20 adolescents each), with some individuals participating on other days to participate and enable us to reach our required sample size. Each question was read aloud to participants, with questions clarified if needed, by the first author. The respondents were given a minute to write down their responses to each statement. The research assistants helped those adolescents who independently filled in the questionnaire to ensure that they had understood all questions and completed the questionnaire. The four adolescents who had writing and hearing problems, respectively, were helped by the research assistants present at each group sitting. The likelihood of experiencing bias in the results are minimal because the ALHIV were required to tick most of the responses in the questionnaire.

### Statistical Data Analysis

Quantitative data were captured from the completed paper questionnaires into a REDCap electronic data entry form. REDCap is a secure, web-based application designed to support research data capture, and provides space for validated data entry and automated export procedures to STATA version 14.2 in readiness for analyses (Harris et al., 2009). Descriptive analysis was performed on demographic and psychometric measures to summarize characteristics of participants. Minimum, maximum, and ranges were reported for all relevant variables. Medians and interquartile range were reported for skewed continuous variables, and means and standard deviations for continuous and normally distributed variables. Frequencies and percentages were used to describe the categorical variables.

Reliability measures like Cronbach’s alpha were used to assess the internal consistency of the resilience measurement scale. A Cronbach’s alpha ≥ 0.70 indicates satisfactory reliability (Tavakol and Dennick, 2011). Kaiser–Meyer–Olkin (KMO) measure and Bartlett’s tests of sphericity were used to assess the adequacy of our sample size and inter-correlations between items to warrant factor analysis (Govender et al., 2017). A KMO closer to 1.0 confirms the feasibility for factor analysis (Acock, 2013; Pritzker and Minter, 2014).

**FIGURE 2:**
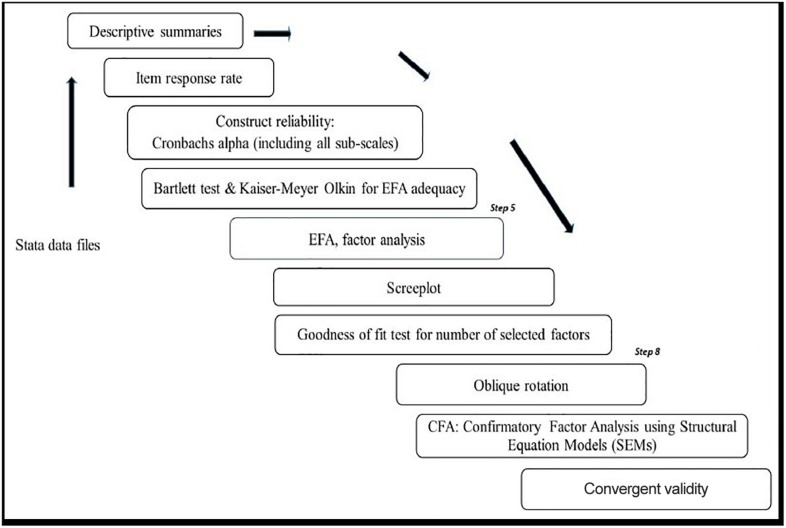
Summary of data analysis process.

The reasons EFA was used in this study include (1) to ascertain the factor structure of the resilience measurement scale, inclusive of all sub-scales (individual, caregiver, and context) to ensure that all items in the scale measured resilience as an underlying construct (Langham et al., 2018) and (2) because our questionnaire items were not normally distributed but were skewed. The CFA using structural equation modeling (SEM) in Stata 14.2 assessed the relationships and inter-relationships between different latent factors of the CYRM-28 of observed and measured behaviors (Acock, 2013). In addition, the CFA determines how the resilience measurement scale conformed to the original conceptual framework (Goretzko et al., 2019). Steps are set out in Figure 2.

The maximum-likelihood estimation method was used to estimate the CFA model. Multiple indices were used to assess the model fit: root mean square error of approximation (RMSEA), Akaike’s Information Criterion (AIC), Bayesian Information Criterion (BIC), Tucker–Lewis Index (TLI), Comparative Fit Index (CFI), and Standardized Root Mean Squared Residual (SRMR) (Govender et al., 2017). Values greater than 0.95 indicate excellent fit to the data whereas values greater than 0.90 indicate acceptable fit to the data (de Carvalho and Chima, 2014). In addition, the average variance extracted (AVE) was estimated to evaluate convergent validity with values greater than 0.50 indicating convergent validity as part of exhibiting evidence in full (Sawatzky et al., 2017).

## Results

A total of 406 ALHIV aged 15–19 years participated, with almost equal numbers of males and females. Their mean age was 16.74, SD 1.46. There was heterogeneity between ethnic and religious groups, although the dominant ethnic groups were Lhlomwe, Chewa, and Ngoni (64% of total participants). Only 3.0% ALHIV did not know their tribe. Religious affiliation, school attendance, access to meals per day, and sleeping spaces varied (Table 1).

**TABLE 1:** Summary of socio-demographic characteristics of participants.

**Variable**	**Overall N (%)**	**Male n (%)**	**Female n (%)**
	406 (100)	204 (50.2)	202 (49.8)
Mean (SD)	1.5(0.50)	1.48(0.50)	1.55(0.40)
**Age**			
**15–16**	196 (48.28)	91 (44.61)	105 (51.98)
**17–19**	210(51.72)	97 (48.02)	113(55.39)
**Education**			
None	38 (9.45)	19(50)	19(50)
Primary	141(35.07)	82(58.16)	59(41.84)
Secondary	223(55.47)	102(45.74)	121 (54.26)
**Ethnic Group**			
Chewa	80 (19.70)	42 (52.50)	38(47.50)
Tonga	18 (4.43)	10(55.56)	8 (44.44)
Sena	26 (6.40)	15(42.32)	11(57.68)
Lhlomwe	101 (24.88)	45(44.55)	56(55.45)
Ngoni	79 (19.46)	43(54.43)	36(45.57)
Tumbuka	40(9.85)	20(50)	20(50)
Yao	34 (8.37)	17(50)	17(50)
Mang’anja	12 (2.96)	4(33.33)	8(66.67)
Don’t Know	12 (2.96)	6(50)	6(50)
Other	4 (0.99)	2(50)	2(50)
**Religion**			
Protestants	205 (50.49)	108(52.68)	97(47.32)
Catholics	83 (20.44)	38(45.78)	45(54.22)
Moslems	28 (6.90)	17(60.71)	11(39.29)
Pentecostals	86 (21.18)	39(45.35)	47(54.65)
Other	4 (0.99)	2(50)	2(50)
**Ever had a baby**			
Yes	12 (2.9)	3(1.5)	9(4.5)
No	394(97)	201(98)	193(95)
**Death in the family**			
**Yes**	253(63.3)	129(64.2)	124(62.3)
**No**	147(36.57)	72(35.8)	75(37.7)
**Person who died**			
Mother	78(31.2)	35(27.6)	43(34.9)
Father	98(39.2)	52(40.9)	46(37.4)
Brother	24(9.6)	15(11.8)	9(7.3)
Sister	19(7.6)	9(7.0)	10(8.3)
Grandparent	4(2)	3(2.3)	1(1)
Aunt	18(7.2)	11(8.7)	7(5.7)
**Ever moved houses**			
Yes	207(50.9)	104(50.2)	103(49.8)
No	199(49.0)	100(50.3)	99(49.8)
**Frequency of changing homes**			
Once	63(30.7)	31(49.2)	32(50.7)
Twice	53(25.9)	25(47.2)	28(52.8)
Three or more times	67(32.7)	37(55.2)	30(44.8)
I do not know	19(9.3)	8(42.1)	11(57.9)
Other	3(1.5)	2(66.6)	1(33.3)
**Sleeping spaces**			
Alone	167 (41.2)	91(54.5)	76(45.5)
With 2 or three people	120 (29.6)	61(50.8)	59(49.2)
More than 4 people	84 (20.7)	36(42.9)	48(57.4)
I do not know	24 (5.9)	12(50)	12(50)
Other	10 (2.5)	4(40)	6(60)
**Number of meals per day**			
One meal	11(2.7)	6(54.6)	5(45.5)
Two meals	47(11.6)	29(61.7)	18(38.3)
Three meals or more	322(79.5)	155(48.1)	167(51.2)
I do not know	19(4.7)	11(57.8)	8(42.1)
Other	6(1.5)	5(50)	3(50)

Overall, the median score for resilience was 123, with an interquartile range of 110–130. The minimum and maximum scores were 53 and 140, with most scores concentrated on the higher side. Five adolescents attained the maximum score of 140 and only one recorded 53. No adolescent scored the lowest possible score of 28. Bartlett’s test of sphericity, 2766.820, *p* < 0.001, showed that our variables had high correlation, which warranted factor analysis. The KMO measure of sampling adequacy was 0.866, confirming that our sample was good enough to continue with EFA (Acock, 2013). The alpha, which is a measure of reliability and showed the internal consistency of the measured dimension, recorded 0.862 for the overall resilience construct whereas the sub-scales showed individual component, α = 0.733; relational component, α = 0.702; and contextual component, α = 0.667. Results in Table 2 show internal reliability for measures of reliability and construct reliability. However, alpha can be high even with items that are minimally related to one another. Therefore, we ran a factor analysis to show correlations between dimensions that represent a shared meaning among the 28-item CYRM measure (Liebenberg et al., 2012; Mampane, 2014).

**TABLE 2:** Alpha reliability.

**Item**	**Sign**	**it-cor**	**is-cor**	**ii-cov**	**alpha**
1. I have people I look up to in my life	+	0.514	0.454	0.29838	0.887
2. I cooperate with people around me	+	0.478	0.417	0.30079	0.888
3. Getting an education is important to me	+	0.511	0.467	0.30401	0.887
4. I know how to behave in different social situations	+	0.482	0.429	0.30285	0.888
5. My parent(s)/caregiver(s) watch me closely	+	0.616	0.581	0.30087	0.886
6. My parents/caregivers know a lot about me	+	0.535	0.489	0.30164	0.887
7. If I am hungry, there is enough to eat at home	+	0.488	0.429	0.30069	0.888
8. I try to finish what I start	+	0.554	0.508	0.30004	0.886
9. Spiritual beliefs are a source of strength for me.	+	0.567	0.519	0.29868	0.886
10. I am proud of my tribe’s background	+	0.550	0.508	0.30227	0.888
11. People think I am funny to be with	+	0.520	0.474	0.30274	0.887
12. I talk to my family/caregiver(s) about how I feel	+	0.528	0.472	0.29878	0.887
13. I can solve problems without harming myself or others by using drugs or violence	+	0.400	0.320	0.3025	0.891
14. I feel supported by my friends	+	0.477	0.408	0.29854	0.889
15. I know where to go in my community to get help	+	0.446	0.367	0.2989	0.890
16. I feel I belong at my school	+	0.535	0.473	0.29556	0.887
17. My family stands by me during difficult times.	+	0.519	0.463	0.29898	0.887
18. My friends stand by me during difficult times.	+	0.472	0.402	0.29886	0.889
19. I am treated fairly in my community	+	0.531	0.466	0.2951	0.887
20. I have opportunities to show others that I am becoming an adult and can act responsibly	+	0.539	0.485	0.29826	0.887
21. I am aware of my own strengths	+	0.538	0.494	0.30225	0.888
22. I participate in organized religious activities	+	0.586	0.539	0.29698	0.886
23. I think it is important to serve my community	+	0.530	0.581	0.30097	0.887
24. I feel safe when I am with my family/caregivers.	+	0.531	0.487	0.30259	0.887
25. I have opportunities to develop skills useful in life, like job skills and skills to care for others).	+	0.499	0.441	0.30013	0.888
26. I enjoy my family’s/caregiver’s cultural and family traditions	+	0.511	0.460	0.30142	0.887
27. I enjoy my community’s tradition	+	0.514	0.452	0.29237	0.888
28. I am proud to be Malawian	+	0.403	0.369	0.31258	0.889
**Test scale**				0.30045	0.891* mean

The EFA through factor analysis showed three factors with eigenvalues above 1. The eigenvalues were obtained for each factor to identify a measure of variance. The first component showed an eigenvalue of 5.63, out of 28 items, and explained 63% of the variance. The second and third factors recorded 1.41 and 1.1 eigenvalues explaining 79% variance of the second factor, whereas 92% explained the total variability of the three factors. In addition, we used the scree plot to confirm the three points of inflection for the final analysis (Ledesma et al., 2015).

Consistent with previous studies on the conceptualization of resilience as a dynamic process and a multi-dimensional construct over time (Liebenberg et al., 2012; Govender et al., 2017; van Rensburg et al., 2019), we computed the factor analysis with oblique rotation (direct oblimin) to accentuate and show the rotated correlations on the three components (individual, relational, and contextual) that make up the resilience measure. Two out of 28 items (items 1 and 15) did not load: “I have people I look up to” and “I know where to go in my community to get help.” Four items of the CYRM-28 had factor loadings <0.350 (items 11, 13, 17, and 28) showing inconsistencies on the role of individual and contextual components in informing resilience across cultures.

We again performed factor analysis on the three-factor model with eigenvalues greater than 1. Factor loadings in the three-factor solution showed that 15 items loaded on factor 1, whereas 10 items and 3 items loaded on factor 2 and factor 3, respectively. One item – “I feel supported by friends” – loaded on both factors 1 and 3, and one item – “I am treated fairly in my community” – loaded on both factors 2 and 3. Table 3 shows the three-factor solution under the three sub-scales after rotation. Ten items and seven items loaded on individual and relational components. Nine items out of 10 loaded on the contextual component. Twenty-six items in the three-factor model had good loadings of >0.3, a minimum criterion for threshold loadings (Acock, 2013; Ledesma et al., 2015). Based on theoretical models of resilience, therefore, we adopted a three-factor structure to better explain resilience among ALHIV (Liebenberg et al., 2012; Gale et al., 2013; Pritzker and Minter, 2014).

**TABLE 3:** Pattern matrix of the three-factor solution for the CYRM-28.

**Components**			
	**Individual**	**Relational**	**Context**
1. I have people I look up to in my life			
15. I know where to go in my community to get help			
2. I cooperate with people around me	0.423		
8. I try to finish what I start	0.576		
4. I know how to behave in different social situations	0.553		
11. People think I am funny to be with	0.343		
13. I can solve problems without harming myself or others by using drugs or violence	0.341		
14. I feel supported by my friends	0.498		
18. My friends stand by me during difficult times.	0.440		
20. I have opportunities to show others that I am becoming an adult and can act responsibly	0.447		
21. I am aware of my own strength	0.485		
24. I feel safe when I am with my family/caregiver	0.474		
25. I have opportunities to develop skills useful in life, like job skills and skills to care for others).	0.568		
5. My parent(s)/caregiver(s) watch me closely		0.581	
6. My parents/caregivers know a lot about me		0.499	
7. If I am hungry, there is enough to eat at home		0.547	
12. I talk to my family/caregiver(s) about how I feel		0.417	
17. My family stands by me during difficult times.		0.313	
26. I enjoy my family’s/caregiver’s cultural and family traditions		0.590	
3. Getting an education is important to me			0.649
9. Spiritual beliefs are a source of strength for me.			0.522
10. I am proud of my tribe’s background			0.593
16. I feel I belong at my school			0.336
19. I am treated fairly in my community			0.446
22. I participate in organized religious activities			0.637
23. I think it is important to serve my community			0.511
27. I enjoy my community’s tradition			0.580
28. I am proud to be Malawian			0.327

The internal reliability of sub-scales was re-assessed using alpha and sample adequacy by KMO, which retained similar results, confirming items for the CFA. This allowed the analysis of the sub-scales to show similar results as in the initial EFA model. Hence, the decision to retain all 28 items for a resilience measure was informed by reliability tests, EFA, and a socio-ecological framework that reflects on individual, relational, and contextual interactions and supports to enable resilience over time (van Rensburg et al., 2015; Ungar, 2016).

### Confirmatory Factor Analysis

**FIGURE 3:**
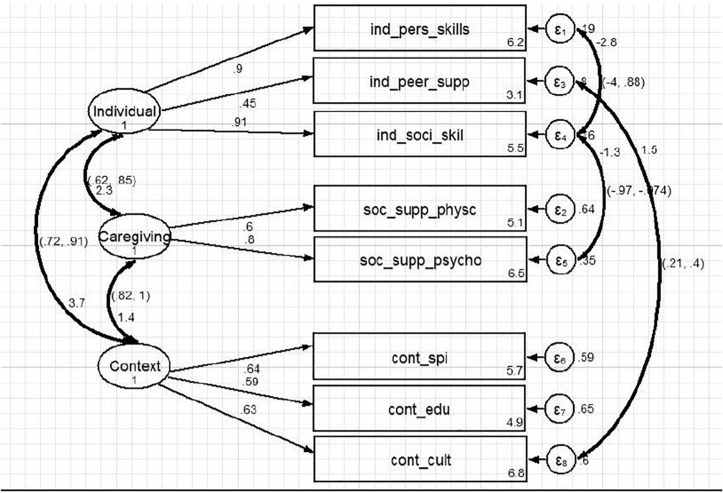
Confirmatory factor analytic model.

The confirmatory factor analysis confirmed the three-factor structure (individual, relational, and contextual) of CYRM-28 with clustered items (see Figure 3).

Statistical methods normally use one statistical test to determine the significance of analysis as well as model fit (Ungar, 2016; Le et al., 2017). Our study employed multiple indices rather than a single index to confirm a model fit to ensure our study results will be comparable at local and international arenas (see Appendix 3) for factor-level analysis and comparable results for our model. The overall fit for the source of resilience model after covariance is illustrated as χ^2^ (*p* < 0.001), df = 1, RMSEA = 0.06, SRMR = 0.03, TLI = 961, CFI = 982, *p* = 0.002, AIC = 14,287.937, and BIC = 14,412.133. There is a need for flexibility when using multiple indices to apply the goodness-of-fit guidelines to inform decision-making in practice (Govender et al., 2017; van Rensburg et al., 2019). Hence, the *p* value, TLI, CFI, SRMR, and RMSEA showed a relatively adequate fit for our model. The interpretability of the fit indices is significant and provided space to understand correlations across and within the sub-scales, including the overall resilience construct.

The factor loadings in the model were positive, and the standardized loadings were high (>0.60) except for individual: peer support (0.45) and contextual: education (0.59). This suggests that all three latent variables were significant and positively correlated in the sample (Liebenberg et al., 2012; Acock, 2013). The CI for the correlation between the corresponding subscales of context and caregiving shows (0.82) and caregiving and individual components recording (0.62, 0.85) and (0.72, 0.91) for context and individual sub-scales. The strong correlations reflect the presence of resilience as an underlying construct. All items, factors, and sub-scales in this Malawian model were statistically significant (*p* ≤ 0.001). Hence, our model confirms that the sub-scales did measure resilience. Composite reliability (CR), and convergent and discriminant validity were performed after fitting the model as part of validating resilience construct in full (Ungar and Liebenberg, 2011). Two dimensions exceeded 0.50 (0.616 individual and 0.50 caregiving), whereas the context recorded 0.38 and had problems with convergent validity. All three constructs had discriminant validity problems (this was expected since all constructs are more related than different (Table 4).

**TABLE 4:** Factor loadings, composite reliability, and average variance extracted.

**Construct/sub-scale**	**Factor loadings**	**Composite reliability (CR)**	**Average variance extracted (AVE)**
Individual		0.817	0.616
• Personal skills	0.90		
• Peer support	0.45		
• Social skills	0.91		
Caregiving		0.665	0.502
• Physical	0.60		
• Psychological	0.80		
Context		0.655	0.387
• Spiritual	0.64		
• Education	0.59		
• Cultural	0.63		

## Discussion

The purpose of this study was to explore the factor structure of the CYRM-28 in a sub-population of ALHIV aged 15–19 and receiving teen-club clinic support in Malawi. This was part of a broader objective to validate the CYRM-28 by assessing the adaptation processes which included slight changes in the wording and translation to vernacular Chichewa. Our study results showed varied perceptions of components and scales contributing to their resilience. In particular, ALHIV perceptions differed on the individual peer support and education supports. This is consistent with previous studies that argue that across sub-populations, there are common resilience elements, but the way they are grouped is different (Langham et al., 2018; Van Breda, 2018). Overall, our study demonstrates construct validity (dependent on measurement scores, (1) content; (2) item responses; and (3) relations to other dimensions, e.g., convergent, discriminant, concurrent (Ungar and Liebenberg, 2011; Pritzker and Minter, 2014), informs gaps in HIV and resilience research (van Rensburg et al., 2015; de Araújo et al., 2017), and highlights the need to be mindful of the role of peers, community, and educational support in the lives of ALHIV. This suggests the value of further qualitative research.

Items 1 and 15, in relation to individual personal and social skills, failed to load in the CYRM-28. This failure of factor loadings is not new; studies using the similar English-language version of CYRM-28 in Canada and South Africa failed on items 1, 5, and 28 (Liebenberg et al., 2012; Govender et al., 2017). The items that failed to load – “I have people I look up to” and “I know where to go in my community to get help” – may reflect the exceptional nature of HIV/AIDS (Moyer and Hardon, 2014). Fear, social exclusion, self-stigma, and social stigma and discrimination continue to affect the everyday lives of people in Malawi, and this ultimately influences people’s interactions with services. However, in line with our theoretical notions on the socio-ecological framework, we retained all statements on CYRM 28-item to measure and understand resilience at multiple levels (Van Breda and Theron, 2018).

In ALHIV research in Malawi, there are mixed outcomes of the role of peers and education in improving well-being and resilience (Amzel et al., 2013; Toska et al., 2016). For instance, Baird et al. (2012) and Toska et al., 2016 have observed that conditional cash transfers linked to school enrollment encourage young people staying in school longer, and in association, this lowered teen pregnancies. However, in our study, some adolescents might have underreported their access to educational support as part of the need for incentives for their education. Low factor loadings were also recorded in previous studies in South Africa, Canada, and Australia on items 1 and 28, which provides more evidence on some of the problematic items that make up the CYRM-28 (Ungar, 2016; Govender et al., 2017; Langham et al., 2018). Our study highlights caregiving support and spiritual and cultural supports as key to informing resilience among ALHIV attending ART clinics in Malawi. Focusing on physical and psychological caregiving, adolescents’ spiritual and cultural supports through religion and rites of passage have been identified as key protective factors for ALHIV (Van Breda and Theron, 2018; OECD Development Centre, 2018).

The results from modeling and resilience demonstrated problems of convergent validity, evidenced by low to moderate correlations between dimensions. The challenge with convergent and discriminant validity may be a result of the fact that the constructs are composed by few indicators and were being assessed in the same model (Byrne, 2012). However, using SEM showed that individual, relational, cultural, and spiritual contexts are closely correlated. The individual personal and social skills work hand in hand with psychological caregiving whereas cultural and individual peer support factors interact for a better fit. The possible explanation for this is that in some cultures, the status of “being an adolescent” may be connected to less prestige and less respect than that of adults. Previous studies on resilience among ALHIV in South Africa have shown the role of community interdependence as illustrated by ubuntu/batho concepts as key factors underlying resilience (Theron and Theron, 2010; Van Breda, 2018). In our study, ALHIV did not identify as a person in and of the community, but rather, they identified as individuals, with less emphasis on peer connectedness and community membership as sources of resilience owing to the stigma and discrimination of living with HIV. In addition, in relation to discrimination, most societies are adult centered, meaning that the needs and preferences of adults are given priority compared with those ones of adolescents whose specific interests are not taken into account. Further qualitative research should elaborate on how ALHIV express and interpret their resilience.

## Implications

Current validation studies on resilience note the need to use the CYRM-28 to identify resilience-enabling sources and resources across cultures and sub-population (Sanders et al., 2015; Masten, 2016; van Rensburg et al., 2019). This study was conducted among adolescents accessing ART and psychosocial support at a teen-club clinic. This study foreshadows the resilience profiles of ALHIV contextually, and so responds to the need to be sensitive on how ALHIV interact with individual, relational, and socio-cultural resources and dynamics as key sources for resilience.

The provision of services under the model of a one-stop shop or the differentiated service delivery approach to meet ALHIV complex needs is key to improving resilience-related outcomes (MacKenzie et al., 2017). Adapting and validating the CYRM-28 for ALHIV in Malawi allowed for the description of various factors or resources that they recognize and interact with for better program planning and implementation. Future research might address the usefulness of the CYRM-28 in measuring intervention outcomes.

### Strength and Limitations

This study provides preliminary evidence on the use of CYRM-28 in Malawi. By adopting the strength-based approach in this study, we looked at ALHIV interacting in meaning and decision-making processes regarding individual, relational, and resourceful supports in their lives. However, this assessment was a once-off process in a single context, among adolescents attending a teen-club clinic. Caution should be applied when extending the results to the general adolescent population. Although we captured resilience among ALHIV with a heterogeneous cultural background, for comprehensive evidence on validation, there is a need to consider adolescents across sub-populations, including those living without HIV, to enrich our findings and to help generalize research and intervention implications.

## Conclusion

Our study contributed to technical and statistical evidence around using CYRM-28 to measure factors and processes underlying resilience among ALHIV in a low-income country. The findings supported the three-factor structure comprising 28 items representing individual, relational, and community/spiritual dimensions. The CYRM is a reliable measure of adolescent resilience, although additional research may be required to validate the instruments among alternative populations and adolescent sub-groups in Malawi.

## Data Availability Statement

All datasets generated for this study are included in the article/Supplementary Material.

## Ethics Statement

The studies involving human participants were reviewed and approved by the Human Research Ethics Committee (HREC) of University of the Witwatersrand, Johannesburg, South Africa and the College of Medicine Research and Ethics Committee (COMREC), Blantyre, Malawi. The ethics clearance numbers for HREC and COMREC are M180465 and P.04/18/2389, respectively, are M180465 and P.04/18/2389, respectively. Written informed consent to participate in this study was provided by the participants’ legal guardian/next of kin. Written informed consent was obtained from the individual(s), and minor(s)’ legal guardian/next of kin, for the publication of any potentially identifiable images or data included in this manuscript.

## Author Contributions

BK-K conceived, designed, and managed the study protocol. BK-K, RD, KM, EM, and LC undertook the field collections and contributed to the write-up on study setting and teen-club programmes. BK-K, IM, RD, KM, AM, EC, SP, and LM focused on both qualitative and quantitative analyses and interpretation. BK-K, IM, and LM conceived, designed, managed, analyzed data, and wrote the manuscript. All authors read, edited, and approved the final version of the manuscript.

## Conflict of Interest

The authors declare that the research was conducted in the absence of any commercial or financial relationships that could be construed as a potential conflict of interest.

## Footnotes

1 www.resilienceresearch.org
