# Supplementary material for: Validating a Child Youth Resilience Measurement (CYRM-28) for Adolescents Living With HIV (ALHIV) in Urban Malawi
Source: Front Psychol. 2020 Aug 31;11:1896. doi: 10.3389/fpsyg.2020.01896 (PMC7488208; doi:10.3389/fpsyg.2020.01896)
Supplement: Supplementary file 1 [file Table_1.DOCX]

**Appendix 1. Site specific questions for ALHIV attending ART clinic**

| Item Context |
| --- |

1 I know what I need to grow up well here Home

2 Adolescents grow up well, here despite the many problems they face Community

3 The family is the main source of support Home

4 Being an adolescent is challenging Individual

5 I am proud of my tribe Home

6 I use health services in my life Hospital

7 I can handle difficulties in my life Individual

8 I use sexual and reproductive services Hospital

9 I have sexual and reproductive health knowledge Individual

10 The teen club provides my support Hospital

11 I am usually happy and healthy Home

|  |
| --- |
