# Supplementary material for: Validating a Child Youth Resilience Measurement (CYRM-28) for Adolescents Living With HIV (ALHIV) in Urban Malawi
Source: Front Psychol. 2020 Aug 31;11:1896. doi: 10.3389/fpsyg.2020.01896 (PMC7488208; doi:10.3389/fpsyg.2020.01896)
Supplement: Supplementary file 2 [file Table_2.DOCX]

**Appendix 2. Adapted CYRM-28 for ALHIV and attending ART clinic**

| **Adapted wording** | **Context** | **Sub-scale** |
| --- | --- | --- |
| 1.I have people I look up to in my life | Home | Context: Cultural |
| 2. I cooperate with people around me | Home | Individual: Personal skills |
| 3. Getting an education is important to me | School | Context: Education |
| 4. I know how to behave in different social situations | Home | Individual: Social Skills |
| 5. My parent(s)/caregiver(s) watch me closely | Home | Caregiver: Physical care |
| 6. My parents/caregivers know a lot about me | Home | Caregiver: Psychological care |
| 7. If I am hungry, there is enough to eat at home | Home | Caregiver: Physical care |
| 8.I try to finish what I start | Home | Individual: Personal skills |
| 9. Spiritual beliefs are a source of strength for me. | Religion | Context: Spiritual |
| 10.I am proud of my tribe’s background | Home | Context: Cultural |
| 11. People think I am funny to be with | Home | Individual: Personal skills |
| 12. I talk to my family/caregiver(s) about how I feel | Home | Caregiver: Psychological care |
| 13. I can solve problems without harming myself or others by using drugs or violence | Home | Individual: Personal skills |
| 14. I feel supported by my friends | Home | Individual: Peer support |
| 15. I know where to go in my community to get help | Community | Individual: Social skills |
| 16. I feel I belong at my school | School | Context: Education |
| 17. My family stands by me during difficult times. | Home | Caregiver: Psychological care |
| 18. My friends stand by me during difficult times. | Home | Individual: Peer support |
| 19.I am treated fairly in my community | Home | Context: Cultural |
| 20. I have opportunities to show others that I am becoming an adult and can act responsibly | Home | Individual: Social skills |
| 21. I am aware of my own strengths | Home | Individual: Personal skills |
| 22. I participate in organized religious activities | Religion | Context: Spiritual |
| 23. I think it is important to serve my community | Community | Context: Spiritual |
| 24. I feel safe when I am with my family/caregivers. | Home | Caregiver: Psychological care |
| 25. I have opportunities to develop skills useful in life, like job skills and skills to care for others). | School | Individual: Social skills |
| 26. I enjoy my family's/caregiver’s cultural and family traditions | Home | Caregiver: Psychological care |
| 27. I enjoy my community’s tradition | Community | Context: Cultural |
| 28. I am proud to be Malawian | Home | Context: Cultural |
