# Supplementary material for: Validating a Child Youth Resilience Measurement (CYRM-28) for Adolescents Living With HIV (ALHIV) in Urban Malawi
Source: Front Psychol. 2020 Aug 31;11:1896. doi: 10.3389/fpsyg.2020.01896 (PMC7488208; doi:10.3389/fpsyg.2020.01896)
Supplement: Supplementary file 3 [file Table_3.DOCX]

**Appendix 3: Summaries of model/Goodness-of-fit for CYRM-28 item scale**

| **Models** | **χ²** | **df** | **RSMEA** | **values** | **CFI** | **TLI** | **SRMR** | **RMSEA [90% CI]** | **AIC** | **BIC** |
| --- | --- | --- | --- | --- | --- | --- | --- | --- | --- | --- |
| **Canadian model (1)*** | **600.229** | **339** | **-** | **-** | **.775** | **-** | **-** | **.057** | **790.229** | **-** |
| **Canadian model (2)**** | **2557.60** | **347** | **-** | **<0.001** | **.832** | **.816** | **.51** | **0.59** | **-** | **-** |
| **South African model (3) ***** | **602.784** | **345** | **0.056** | **-** | **0.778** | **-** | **-** | **-** | **780.784** | **-** |
| **Australian model (4) ****** |  |  |  | **.184** | **.960** |  |  | **.026** | **47.022.06** | **47.411.09** |
| **New Zealand model**  **model (5) ******* | **708.43** | **344** | **.04** | **>.05** | **.87** | **.85** | **.05** | **.04, .05** | **-** | **-** |
| **Malawi model (6)** ********** | **32.188** | **1** | **.06** | **<0.001** | **.982** | **.961** | **.029** | **.03, .09** | **14287.937** | **14412.133** |

Notes

*Model 1: Goodness of fit models (Langham et al. 2018)

**Model 2: Original three-factor model, including all 28 items (Liebenberg et al., 2012; Ungar & Liebenberg, 2011).

*******Model 3: Three-factor model including replaced variables (Langham et al. 2018)

********Model 4: Three-factor model, including all 28 items ((Langham et al. 2018)

*********Model 5: Three-factor model, including all 28 items (van Rensburg et al. 2019).

**********Model 6: Three-factor model, including all 28 items (current study).
